# Highly Pathogenic Clone of Shiga Toxin–Producing *Escherichia coli* O157:H7, England and Wales

**DOI:** 10.3201/eid2412.180409

**Published:** 2018-12

**Authors:** Lisa Byrne, Timothy J. Dallman, Natalie Adams, Amy F.W. Mikhail, Noel McCarthy, Claire Jenkins

**Affiliations:** Public Health England, London, UK (L. Byrne, T.J. Dallman, N. Adams, A.F.W. Mikhail, C. Jenkins);; National Institute for Health Research, London (T.J. Dallman, N. Adams, N. McCarthy);; University of Warwick, Coventry, UK (N. McCarthy)

**Keywords:** Shiga toxin–producing *Escherichia coli*, whole-genome sequencing, virulence, evolution, bacteria, enteric infections, England, Wales, United Kingdom, STEC

## Abstract

We used whole-genome sequencing to investigate the evolutionary context of an emerging highly pathogenic strain of Shiga toxin–producing *Escherichia coli* (STEC) O157:H7 in England and Wales. A timed phylogeny of sublineage IIb revealed that the emerging clone evolved from a STEC O157:H7 *stx-*negative ancestor ≈10 years ago after acquisition of a bacteriophage encoding Shiga toxin (*stx*) 2a, which in turn had evolved from a *stx2c* progenitor ≈20 years ago. Infection with the *stx2a* clone was a significant risk factor for bloody diarrhea (OR 4.61, 95% CI 2.24–9.48; p<0.001), compared with infection with other strains within sublineage IIb. Clinical symptoms of cases infected with sublineage IIb *stx2c* and *stx*-negative clones were comparable, despite the loss of *stx2c.* Our analysis highlighted the highly dynamic nature of STEC O157:H7 Stx-encoding bacteriophages and revealed the evolutionary history of a highly pathogenic clone emerging within sublineage IIb, a sublineage not previously associated with severe clinical symptoms.

Shiga toxin–producing *Escherichia coli* (STEC) O157:H7 cause a wide range of gastrointestinal symptoms, including mild gastroenteritis, abdominal pain, vomiting, and bloody diarrhea (*1*). A subset of patients, most commonly the very old and the very young, go on to develop hemolytic uremic syndrome (HUS) (*2*). STEC O157:H7 are zoonotic, and transmission to humans is most commonly associated with ruminants, especially cattle and sheep. Transmission occurs by consumption of contaminated food or water or by direct contact with animals or their environment. The infectious dose is low (10–100 organisms), and person-to-person spread can occur in households, nurseries, and other institutional settings (*1*). The STEC pathotype is defined by the presence of the genes encoding Shiga toxin (Stx) type 1, type 2, or both, located on a bacteriophage incorporated into the bacterial genome (*3*). Stx1 and Stx2 can be further divided into subtypes Stx1a–1d and Stx2a–2g; Stx2a is strongly associated with causing severe disease (*4**,**5*). The STEC O157:H7 population has previously been delineated into 3 main lineages (I, I/II, and II) (*6*), and 7 sublineages (Ia, Ib, Ic, IIa, IIb, IIc, and I/ll) (*5*).

In England, the most common STEC serotype is O157:H7, which causes an average of 800 cases/year (*1*). All STEC O157:H7 isolated at local hospital laboratories from fecal samples from hospitalized patients and all cases in the community are submitted to the Gastrointestinal Bacteria Reference Unit (GBRU) at Public Health England for confirmation of identification and typing. From 2000 through 2016, phage type (PT) 8 with the *stx* profile *stx1a/stx2c* and PT21/28 with the *stx* profile *stx2a* or *stx2a/stx2c* were detected most frequently in England, with PT21/28 the most frequently associated with severe disease (*2**,**7*).

Since 2015, all isolates submitted to GBRU have been genome sequenced. Whole-genome sequencing (WGS) demonstrates unparalleled sensitivity and accuracy in identifying linked cases (*8*). Using WGS data during outbreak investigations has improved the robustness of case ascertainment and provided forensic evidence for linking human cases to the source of their infection (*9**,**10*). Phylogenetic inference can also reveal how strains are related over time and space more accurately than other molecular typing methods and may provide insight into the evolutionary and epidemiologic context of emerging pathogenic clones (*8**,**10**,**11*).

In 2015, a total of 47 persons were affected by an outbreak in England of foodborne gastrointestinal illness caused by STEC O157:H7 PT8 *stx2a*. The outbreak was associated with the consumption of contaminated prepacked salad leaves (*11*). The outbreak strain continued to cause sporadic infection and outbreaks of foodborne disease throughout 2016 and 2017 (*11*). The aim of our analysis was to investigate the evolutionary history of this newly emergent strain of STEC O157:H7 PT8 *stx2a* and assess the risk to public health.

## Materials and Methods

### Bacterial Strains

All isolates submitted to GBRU for confirmation and typing from local hospital laboratories in England and Wales during July 2015–December 2017 were sequenced for routine surveillance National Center for Biotechnology Information Short Read Archive bioproject PRJNA248042). We included an additional 74 clinical isolates of STEC O157:H7 belonging to sublineage IIb, the lineage containing STEC O157:H7 PT8 *stx2a*, that were submitted to GBRU between January 2010–June 2015 from previous studies (*5**,**8*) (Technical Appendix Table). We selected these STEC O157:H7 isolates on the basis of *stx* subtype and phage type diversity to provide context as a sample of the background population. We defined STEC O157:H7 isolates from patients who were hospitalized as a result of their gastrointestinal symptoms or who reported bloody diarrhea as highly pathogenic or as having increased pathogenic potential compared with isolates from patients who were asymptomatic or reporting nonbloody diarrhea.

### Genome Sequence Analysis

For WGS, we extracted DNA from cultures of STEC O157:H7 for sequencing on the HiSeq 2500 instrument (Illumina, San Diego, California, USA). We mapped quality trimmed Illumina reads (*12*) to the STEC O157:H7 reference genome Sakai (GenBank accession no. BA000007) using Burrows Wheeler Aligner-Maximum Exact Matching (BWA-MEM) (*13*). We identified single-nucleotide polymorphisms (SNPs) using Genome Analysis Toolkit version 2 (*14*) in unified genotyper mode and extracted core genome positions that had a high-quality SNP (>90% consensus, minimum depth ×10, GQ >30) in >1 isolate for further analysis. We performed hierarchical single linkage clustering on the pairwise SNP difference between all strains at various distance thresholds (250, 100, 50, 25, 10, 5, 0). The result of the clustering is a SNP profile, or SNP address, that can be used to describe population structure based on clonal groups (*15*). 

We performed recombination analysis using Gubbins (*16*) and reconstructed timed phylogenies using BEAST-MCMC version 2.4.7 (*17*). We computed alternative clock models and population priors and assessed their suitability on the basis of Bayes factor tests. The highest supported model was a relaxed log-normal clock rate with a Bayesian skyline population model. We ran all models with a chain length of 1 billion. We reconstructed a maximum clade credibility tree using TreeAnnotator version 1.75 (*17*).

We performed Stx subtyping as described by Ashton et al. (*18*). The integration of Stx-encoding prophage into the host genome has been characterized into 6 target genes: *wrbA*, encoding a NAD quinone oxidoreductase; *yehV*, a transcriptional regulator; *sbcB*, an exonuclease; *yecE*, a gene of unknown function; the tRNA gene *argW*; and Z2577, which encodes an oxidoreductase (*5*). We mapped short reads from the STEC O157:H7 genomes to intact reference sequences of these genes, and aligned them with BWA MEM (*13*). We defined occupied Stx bacteriophage insertion (SBI) sites as those strains that had disrupted alignments (*5*). We used Tablet to visualize read pileups (*19*).

### Data Analyses

The National Enhanced Surveillance System for STEC (NESSS) in England was implemented on January 1, 2009, and has been described in detail elsewhere (*1*). For this study, we extracted data from NESSS for the cases identified as being infected with strains that had been sequenced and belonging to the sublineage IIb cluster of interest (containing the STEC O157:H7 PT8 *stx2a*, outbreak strain). We excluded asymptomatic carriers detected through screening high-risk contacts of symptomatic patients as well as patients who did not return the enhanced surveillance questionnaire (ESQ) to NESSS. Data analyzed included age, gender, and whether the patient reported symptoms of nonbloody diarrhea, bloody diarrhea, and vomiting along with whether cases were hospitalized, developed typical HUS, or died. Cases were categorized into children (<16 years of age) or adults, based on a priori knowledge that children are most at risk for both STEC infection and progression to HUS (*1*). Where clinical symptoms were blank on the ESQ, we coded them as negative responses for these symptoms. We divided cases into 3 groups based on *stx* subtype: *stx2a*, *stx2c,* and *stx-*negative.

We first described patients’ symptoms by *stx* subtype as well as by age group and sex and also examined the distribution of *stx* subtype by age and gender. We used Fisher exact tests to compare proportions among different groups. We assessed reporting of bloody diarrhea or hospitalization as a marker of disease severity by *stx* subtype. We used logistic regression to calculate odds ratios (ORs) to assess bloody diarrhea by *stx* subtype while adjusting for age (child/adult) and sex. We performed all analyses in Stata 13.0 (StataCorp LLC, College Station, TX, US).

## Results

### Sublineage IIb

**Figure 1:**
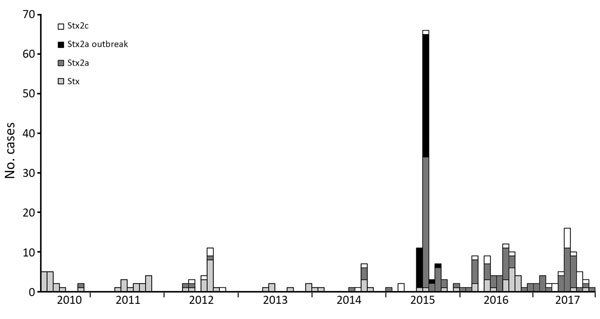
Cases of Shiga toxin–producing *Escherichia coli* O157:H7 belonging to sublineage IIb, 250 single-nucleotide polymorphism single linkage cluster 18.%, by *stx* subtype profile, submitted to the Gastrointestinal Bacterial Reference Unit at Public Health England from England and Wales during June 1, 2010–December 31, 2017.

The STEC O157:H7 *stx2a* clone analyzed in this study was located within sublineage IIb, and belonged to a 250 SNP single linkage cluster, designated 18%. This cluster comprised 251 clinical isolates: 138 of STEC O157:H7 *stx2a*, 77 of *stx-*negative *E. coli* O157:H7, and 36 of STEC O157:H7 *stx2c* (Figure 1; Technical Appendix Table). Since July 2015, when Public Health England implemented the use of WGS for STEC, the number of cases identified within sublineage IIb has remained stable (≈60/y). However, the number of cases of the *stx-*negative *E. coli* O157:H7 clone has declined, whereas the *stx2a* and *stx2c* clones are increasing (Figure 1).

### Evolutionary Timescale and Stx Prophage Insertion in STEC O157:H7

**Figure 2:**
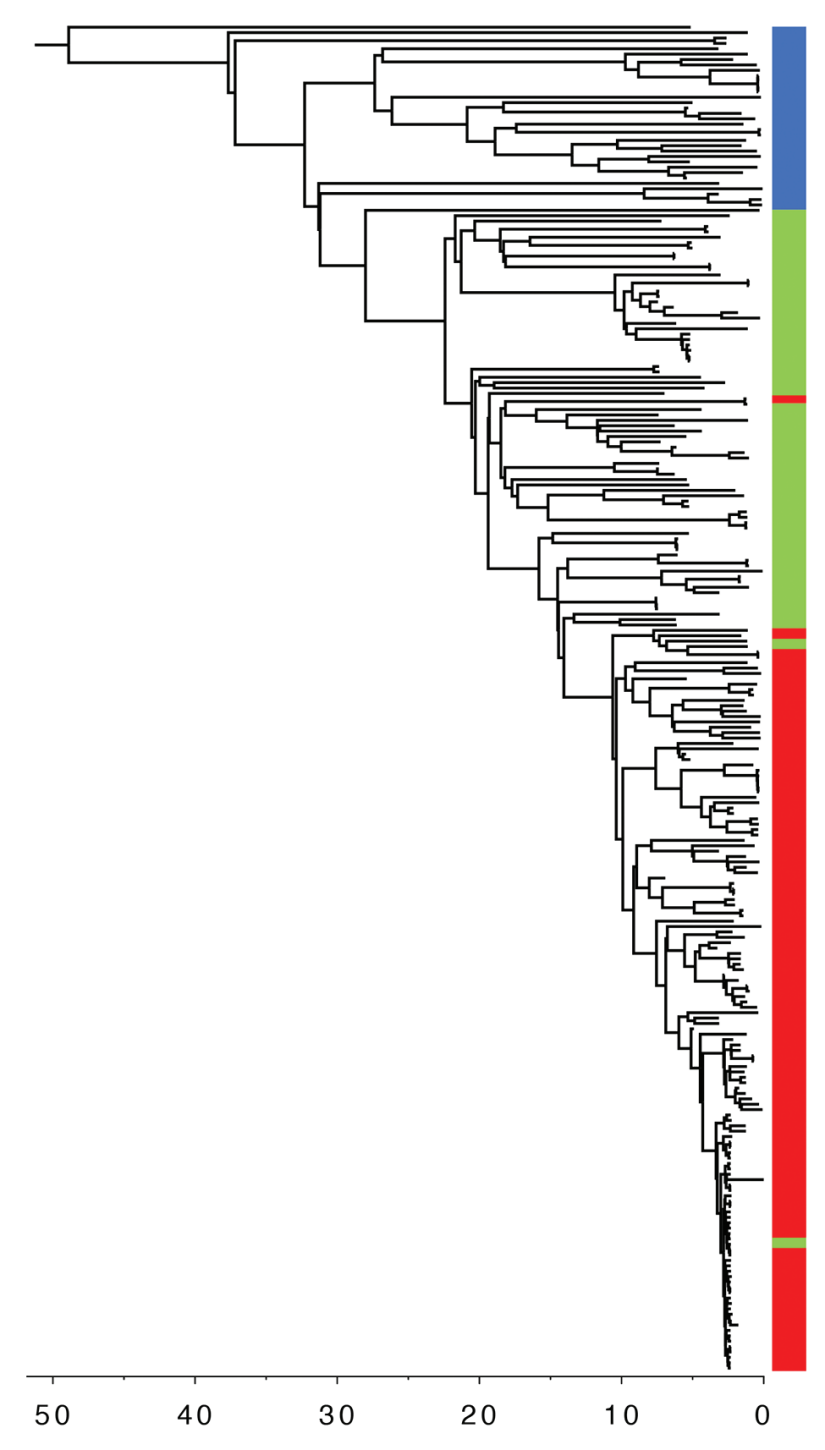
Timed phylogeny of Shiga toxin–producing *Escherichia coli* O157:H7 sublineage IIb isolates illustrating the sequential loss of stx2c and subsequent gain of *stx2a*. Scale bar indicates years in the past.

We reconstructed a timed phylogeny of sublineage IIb (Figure 2). We calculated the mutation rate of STEC O157:H7 within sublineage IIb to be ≈2 mutations per genome per year (95% highest posterior density [HPD] 1.7–2.4). This rate is less than the 2.6 mutations per genome per year previously calculated across the complete STEC O157 population (*5*). Our analysis revealed that the emerging *stx2a* clone evolved from a *stx-*negative recent ancestor with the acquisition of *stx2a* ≈10 years ago (95% HPD 9.0 years–12.7 years). Previously, this *stx*-negative clone had evolved from a *stx2c* progenitor ≈20 years ago (95% HPD 17.6 years–24.6 years) after the loss of *stx2c*.

Historically, the majority of strains in sublineage IIb harbored a Stx2c-encoding prophage at *sbcB*, with the *yehV* SBI site occupied by a truncated non–Stx encoding prophage (*5*). Analysis of the short read data indicated that in the *stx-*negative sublineage IIb clone, *yehV* was disrupted but *sbcB* was intact, indicating the loss of the Stx2c-encoding prophage from the SBI site. The more recently emerged sublineage IIb *stx2a* clone had disrupted SBI sites at *sbcB* and *yehV* only, indicating that a Stx2a-encoding phage had been inserted into *sbcB*, the site left vacant in the *stx-*negative clone after the loss of *stx2c*.

### Disease Severity of Clinical Cases within the Sublineage IIb Cluster by *stx* Subtype

Overall, 91.6% patients (230/251, 95% CI 88.1–95.1) had symptoms of diarrhea, and similar percentages were reported regardless of the *stx* subtype profile of the STEC O157:H7 causing the infection (Table 1). Rates of other symptoms varied; 28.3% of patients (71/251, 95% CI 22.7–33.9) reported vomiting, 35.1% (88/251, 95% CI: 29.1–41.0) experienced bloody diarrhea, and 18.7% (47/251, 95% CI: 13.9–23.5) were hospitalized. Hospitalization occurred more often for patients reporting bloody diarrhea (35.2% [31/88, 95% CI 25.0–45.4]) than those without bloody diarrhea (9.8% [16/163], 95% CI 5.2–14.1; p<0.001). Half (50.0%) of patients infected with *stx2a* isolates reported bloody diarrhea (69/138, 95% CI 41.5–58.4), compared with 15.6% of patients infected with *stx*-negative isolates (12/77, 95% CI 7.3–23.9) and 19.4% of those infected with *stx2c* isolates (7/36, 95% CI 5.9–33.0; p<0.001). No patients were known to experience HUS, and none died.

**Table 1:** Clinical features of cases of Shiga toxin–producing *Escherichia coli* O157:H7 belonging to the IIb 250 SNP single linkage cluster 18.% by *stx* subtype clone, England and Wales, July 2015–December 2017

*stx* subtype	No. patients	Diarrhea			Bloody diarrhea†			Vomiting			Hospitalization‡	
		No. patients	% Patients (95% CI)		No. patients	% Patients (95% CI)		No. patients	% Patients (95% CI)		No. patients	% Patients (95% CI)
*stx2c*	36	31	86.1 (74.2–98.0)		7	22.6 (5.9–33.0)		10	32.3 (12.4–43.1)		3	8.3 (−1.1 to 17.8)
*stx*-negative	77	72	93.5 (87.8–99.1)		12	16.7 (7.3–23.9)		23	31.9 (19.4–40.3)		9	11.7 (4.3–19.0)
*stx2a*	138	127	92.0 (87.4–96.6)		69	54.3 (41.5–58.4)		38	29.9 (20.0–35.1)		35	25.4 (18.0–32.7)
Total	251	230	91.6 (88.1–95.1)		88	38.3 (29.1–41.0)		71	30.9 (22.7–33.9)		47	18.7 (13.9–23.5)

*SNP, single-nucleotide polymorphism. †Statistically significant difference in reporting by *stx* subtype using Fisher exact test (p<0.001) ‡Statistically significant difference in reporting by *stx* subtype using Fisher exact test (p = 0.011)

Among the 251 clinical cases, 141 (56.2%, 95% CI 50.0%–62.3%) were adults and 136 (54.2%, 95% CI 48.0%–60.4%) were female. Adult patients were infected with *stx2a* strains (61.0% [86/141], 95% CI 52.8%–69.1%) more often than children (47.3% [52/110], 95% CI 37.8%–56.7%; p = 0.030). Conversely, children were more often infected with *stx-*negative strains than adults: 41.8% (46/110) of children (95% CI 32.4%–51.2%) versus 22.0% (31/141) of adults (95% CI 15.1%–28.9%; p = 0.001). There was also variation in *stx* subtype by sex; proportionately more female patients were infected with *stx2a* strains (61.0% [83/136], 95% CI 52.7%–69.3%) than were male patients (47.8% [55/115], 95% CI 38.6%–57.1%; p = 0.036). Adult patients reported bloody diarrhea (46.8% [66/141], 95% CI 38.5%–55.1%) more often than children (20.0% [22/110], 95% CI 12.2%–27.6%; p<0.001), as did female patients (40.4% [55/136], 95% CI 32.1%–48.8%) compared with male patients (28.7% [33/115], 95% CI 20.3%–37.1%), although the difference was not statistically significant (p = 0.05). The proportion of patients hospitalized did not differ significantly by sex or age group (data not shown).

After adjusting for age (adult or child) and sex, the odds ratio of experiencing bloody diarrhea was significantly higher in those infected with the *stx2a* clone compared with patients infected with the *stx*-negative clone (Table 2). The odds of bloody diarrhea were no different for cases infected with the *stx2c* clone than for the *stx*-negative clone. Among the cases analyzed, being a child was protective for symptoms of bloody diarrhea.

**Table 2:** Univariable and multivariable logistic regression analysis for reported bloody diarrhea in cases of Shiga toxin–producing *Escherichia coli* O157:H7 belonging to the IIb, 250 SNP single linkage cluster 18.% by *stx* subtype clone, England and Wales, July 2015–December 2017*

Exposure	No. (%) patients with bloody diarrhea	No. (%) patients without bloody diarrhea					
			Univariable analysis			Multivariable analysis†	
			OR (95% CI)	p value		OR (95% CI)	p value
*stx* subtype							
* stx* negative	12 (16.7)	65 (83.3)	Reference			Reference	
* stx2c*	7 (22.6)	29 (77.4)	1.31 (0.47–3.7)	0.61		1.01 (0.35–2.94)	0.978
* stx2a*	69 (54.3)	69 (45.7)	5.42 (2.69–10.91)	<0.001		4.61 (2.24–9.48)	<0.001
Age							
Child, <16 y	22 (20.0)	88 (80.0)	0.28 (0.16–0.50)	<0.001		0.31 (0.17–0.58)	<0.001
Adult, >16 y	66 (46.8)	75 (53.2)	Reference			Reference	
Sex							
F	55 (40.4)	81 (59.6)	Reference			Reference	
M	33 (28.7)	82 (71.3)	0.59 (0.35–1.01)	0.053		0.81 (0.45–1.47)	0.494

*OR, odds ratio; SNP, single-nucleotide polymorphism.  †Adjusted for each exposure variable (*stx* subtype, sex, age).

## Discussion

The data described here support previous studies that showed the acquisition and loss of the Stx-encoding phage is highly dynamic in STEC O157:H7 (*5**,**20*). Most commonly described is the acquisition of *stx1a* or *stx2a* by a STEC O157:H7 *stx2c* progenitor, followed by the subsequent loss of *stx2c* in strains that acquired *stx2a*. The involvement of a *stx*-negative intermediate in this process, as captured here, has not been previously described. The loss of the Stx2c-encoding phage appears to have facilitated the acquisition of the Stx2a-encoding phage because the latter was inserted into the same SBI site, *sbcB*, left vacant by the Stx2c-encoding phage.

Using phylogenetic analysis of variation at the whole-genome level, we reconstructed the recent evolutionary history of this emerging pathogenic clone within STEC O157:H7 sublineage IIb. We observed the loss of *stx2c* from the *stx2c* progenitor that caused a *stx*-negative clone ≈20 years ago, followed by the acquisition of *stx2a* ≈10 years ago, and later expansion as shown in Figure 1. Previously, we showed that the historic acquisition of a Stx2a-encoding bacteriophage by a population of STEC O157:H7 PT2 *stx2c*, belonging to lineage I/II indigenous in the UK cattle population, was associated with the first outbreaks of childhood HUS in England in the early 1980s (*5**,**7*). Subsequently, the increase in the incidence of STEC O157:H7 PT21/28 during the 1990s was linked to the acquisition of *stx2a* by an indigenous population of STEC O157:H7 *stx2c* belonging to sublineage Ic, resulting in the highly pathogenic contemporary clone STEC PT21/28 *stx2a*/*stx2c* (*1**,**2**,**5**,**7*). This clone has been associated with several outbreaks in the United Kingdom associated with a high incidence of HUS (*10**,**21**–**23*). Here, we described an *E.coli* O157:H7 clone from yet another UK domestic lineage (sublineage IIb) that has recently acquired the Stx2a-encoding phage and is showing evidence of increasing pathogenic potential.

The analysis of disease severity of clinical cases by *stx* subtype of isolates of STEC O157:H7 within the same sublineage IIb cluster showed a significant association between the presence of *stx2a* and markers of disease severity; specifically, bloody diarrhea linked to higher rates of hospitalization. Previous studies have reported evidence of increased pathogenicity of STEC harboring *stx2a* (*4**,**5*). However, these studies report on STEC from a wide range of different serotypes, exhibiting a wide variety of *stx* subtypes and are based on relatively small datasets. In this study, we present the analysis of a large dataset focusing on a specific clade within a single serotype characterized by a limited number of *stx* subtype combinations, specifically *stx2c*, *stx* negative, and *stx2a* only. This analysis enabled us to make direct comparisons between specific *stx* profiles while limiting the influence of other factors in the genome.

Strains of Stx-negative *E. coli* O157:H7 are regarded as atypical enteropathogenic *E. coli* (EPEC), defined by the presence of the intimin gene (*eae*) and the absence of *stx* and the *E. coli* adherence factor (EAF) plasmid (*24*). EPEC are a common cause of infantile diarrhea and travelers’ diarrhea and are known to cause mild diarrhea in adults (*25*). In this study, the fact that clinical cases infected with the *E. coli* O157:H7 *stx*-negative clone reported a similar frequency of symptoms, including bloody diarrhea and hospitalization, as those infected with STEC O157:H7 *stx2c* despite the loss of *stx* was an unexpected finding that requires further investigation.

A timed phylogenetic reconstruction of the evolutionary history of a cluster of sublineage IIb charted the recent emergence of a highly pathogenic clone of STEC O157:H7 *stx2a*. The symptom of bloody diarrhea, a marker of severity and predictor of HUS development (*2*), was strongly associated with cases infected with isolates of STEC O157:H7 harboring *stx2a* compared with those isolates without *stx* or those with *stx2c*. Our analysis also illustrated the highly dynamic nature of the Stx-encoding phages. In contrast to the observed excision events of *stx2c*-encoding phages in O157:H7, there is evidence to suggest that once a Stx2a-encoding phage is integrated into a population it tends to be maintained (*5*). As such, the emergence of yet another sublineage of STEC O157:H7 acquiring *stx2a* is of public health concern. Through this study, we demonstrate that STEC O157:H7 WGS surveillance data have a role in monitoring and anticipating emerging threats to public health and in contributing to our understanding of the underlying pathogenic mechanisms associated with severe gastrointestinal illness.

Technical Appendix Additional information about highly pathogenic clone of Shiga toxin–producing *Escherichia coli* O157:H7.
